# Community drinking water data on the National Environmental Public Health Tracking Network: a surveillance summary of data from 2000 to 2010

**DOI:** 10.1007/s10661-019-7710-y

**Published:** 2019-08-11

**Authors:** Michele M. Monti, Felicita David, Mikyong Shin, Ambarish Vaidyanathan

**Affiliations:** 1 Environmental Health Tracking Section, Division of Environmental Health Science and Practice, National Center for Environmental Health, Centers for Disease Control and Prevention, Atlanta, GA USA; 2 Health Systems and Trauma Systems Branch, Division of Unintentional Injury Prevention, National Center for Injury Prevention and Control, Centers for Disease Control and Prevention, Atlanta, GA USA

**Keywords:** Environmental health tracking·community, Drinking water,Community water systems, Contaminants,·Environmental hazards

## Abstract

This report describes the available drinking water quality monitoring data on the Centers for Disease Control and Prevention (CDC) National Environmental Public Health Tracking Network (Tracking Network). This surveillance summary serves to identify the degree to which ten drinking water contaminants are present in finished water delivered to populations served by community water systems (CWS) in 24 states from 2000 to 2010. For each state, data were collected from every CWS. CWS are sampled on a monitoring schedule established by the Environmental Protection Agency (EPA) for each contaminant monitored. Annual mean and maximum concentrations by CWS for ten water contaminants were summarized from 2000 to 2010 for 24 states. For each contaminant, we calculated the number and percent of CWS with mean and maximum concentrations above the maximum contaminant level (MCL) and the number and percent of population served by CWS with mean and maximum concentrations above the MCL by year and then calculated the median number of those exceedances for the 11-year period. We also summarized these measures by CWS size and by state and identified the source water used by those CWS with exceedances of the MCL. The contaminants that occur more frequently in CWS with annual mean and annual maximum concentrations greater than the MCL include the disinfection byproducts, total trihalomethanes (TTHM), and haloacetic acids (HAA5); arsenic; nitrate; radium and uranium. A very high proportion of exceedances based on MCLs occurred mostly in very small and small CWS, which serve a year-round population of 3,300 or less. Arsenic in New Mexico and disinfection byproducts HAA5 and TTHM, represent the greatest health risk in terms of exposure to regulated drinking water contaminants. Very small and small CWS are the systems’ greatest difficulty in achieving compliance.

## Introduction

A safe water supply is crucial to public health and plays a critical role in our well-being as well as the success of our society and economy (EPA 2016). The USA has one of the safest drinking water supplies in the world (EPA 2016; CDC 2014). Nevertheless, improper disposal of chemicals, animal and human wastes, wastes injected underground, pharmaceuticals, personal care products, and naturally occurring substances have the potential to contaminate drinking water.

Ninety percent of the American public gets their drinking water from a public water system (PWS) (EPA 2017a). By definition, PWS provides water to at least 25 people and have at least 15 service connections (EPA 2017a); community water systems (CWS) are PWS that supply water to the same population year-round (EPA 2017a). There are approximately 151,000 PWS of which 52,000 are CWS (EPA 2017a, 2017b). Eight percent of these systems serve more than 82% of the total population of the USA. Similarly, more water systems use groundwater (78%) rather than surface water as a source, but more people (68%) receive their water from a system supplied by surface water (EPA 2009a). PWS are regulated by the U.S. Environmental Protection Agency (EPA) and state agencies under the Safe Drinking Water Act (SDWA) (EPA 2017c). The National Primary Drinking Water Regulations, or primary standards, protect public health by setting limits on levels of contaminants in drinking water. Those limits are referred to as maximum contaminant levels (MCLs) (EPA 2017b). MCLs have been identified for over 90 drinking water contaminants (EPA 2017c).

Drinking water that is not properly treated or disinfected, or that travels through an improperly maintained distribution system, may pose a health risk (EPA 2009b). There is a broad range of health effects associated with exposure to drinking water contaminants. Ingestion or exposure to pathogens at sufficient doses can result in gastrointestinal illness with symptoms such as diarrhea, nausea, stomach cramps and vomiting, headaches, and other symptoms. Exposure to chemicals, metals, or radionuclides can produce biological responses, toxicological effects, and more severe health impacts including cancer, developmental or reproductive effects, neurological effects, and organ damage (EPA 2011a, 2011b).

The National Environmental Public Health Tracking Program (Tracking Program) was created by Congress to provide information from a nationwide network of health and environmental data that can be used to take action to reduce and prevent disease caused by exposure to environmental health threats (CDC 2018). In 2002, CDC began the task of developing the National Environmental Public Health Tracking Network (Tracking Network) to integrate data on environmental hazards, exposures, and human health conditions (CDC 2018). Chronic conditions such as asthma, heart attacks, birth defects, cancer, childhood lead poisoning, and reproductive outcomes related to environmental hazards and/or exposures lessen the quality of life and contribute to the continuously rising cost of health care (EPA 2009c). The Tracking Network can be used in environmental public health to monitor trends, identify populations or areas at-risk, generate hypotheses, and inform interventions or policies.

The drinking water data used by the Tracking Program are gathered as part of the water quality monitoring requirements established by the EPA and state agencies under the Safe Drinking Water Act. These data, although only for CWS in 24 states funded by the Tracking Program, are the most complete set of concentration data for the 10 drinking water contaminants selected. The ten contaminants selected for the Tracking Network were identified as priority contaminants by a workgroup with representatives from state and local health departments and environmental departments, CDC, and EPA. Annual mean and maximum concentrations of arsenic, nitrates, disinfection byproducts (total trihalomethanes (TTHM) and haloacetic acids (HAA5)), atrazine, DEHP (di (2-ethylhexyl) phthalate), trichloroethylene (TCE), perchloroethylene (PCE), uranium, and radium are displayed on the national public portal of the Tracking Network. These contaminants were selected because of their association with chronic disease health outcomes, and because they occur more often and in the greatest concentrations of all drinking water contaminants monitored by EPA during the Six-Year Review (EPA 2018a). Although lead is a contaminant of interest and concern, lead data are not yet available for inclusion in the Tracking Network. The data presented here are finished water (after treatment) measurements of the 10 contaminants selected for examination. The annual mean and annual maximum measurements are a proxy for exposure of the population served by the CWS. All CWS in a state are represented; water treatment at the different CWS will differ but that is not the focus of this article. This article summarizes the nature and quality of finished drinking water from CWS in 24 states for 10 contaminants. These 10 contaminants are the only drinking water contaminants available from the Tracking Network.

Arsenic contamination in drinking water can result from both natural and man-made activities (EPA 2018b) Arsenic is a naturally occurring element that is deposited in groundwater and surface water from the dissolution of geologic formations, from runoff and leaching of soils, volcanic activity, and forest fires (ATSDR 2007; EPA 2018b). Arsenic is used for wood preservation, in paints, drugs, dyes, soap, metals, and in agricultural and mining applications (EPA 2018b). Arsenic in drinking water has been associated with heart disease, neurological disorders, and cancer of the skin, lung, kidney, and bladder (ATSDR 2007; National Research Council 2001; Martinez et al. 2011).

Atrazine is an herbicide used on agricultural crops to control broadleaf and grassy weeds (EPA 2018c). It is also used on residential lawns and golf courses (EPA 2018c). Exposure to atrazine has been associated with ovarian and thyroid cancer and non-Hodgkin’s lymphoma and hairy-cell leukemia (EPA 2011c).

DEHP is the most commonly used phthalate or phthalic acid ester, which are chemicals used to make plastics more flexible (EPA 2018c; ATSDR 2011). Drinking water containing DEHP in excess of the MCL may lead to health problems associated with the liver, reproductive difficulties, or cancer (EPA 2018d).

Disinfection byproducts (DBP) are formed when disinfectants used to inactivate microbial contaminants in water react with organic matter in the water (Sadiq and Rodriguez 2004; EPA 2018e). DBPs have been associated with cancer; adverse pregnancy outcomes, and reproductive implications (Villanueva et al. 2015; Li and Mitch 2018; Mashau et al. 2018). High DBP levels, mainly for TTHM, have been linked to bladder, colon and rectal cancer (Li and Mitch 2018; Villanueva et al. 2007) with bladder cancer reported most frequently.

Nitrogen-based fertilizers, animal wastes, and malfunctioning septic or sewer systems are all possible sources of nitrates in drinking water (ATSDR 2015). Nitrate was first identified as a public health threat in drinking water in 1945 when high nitrate levels from private wells were shown to cause methemoglobinemia or “blue baby syndrome” in infants who received formula made from well water. The blue baby syndrome is potentially fatal (Ward et al. 2018; EPA 2018f). Exposure to nitrate in drinking water is also associated with adverse reproductive outcomes such as spontaneous abortions, intrauterine growth retardation, and various birth defects such as anencephaly, related to fetal exposures to nitrate (Manassaram et al. 2006). A recent review of the health effects of exposure to nitrate in drinking water found strong evidence for a relationship between drinking water nitrate ingestion and colorectal cancer, thyroid disease, and neural tube defects (Ward et al. 2018).

Tetrachloroethylene (PCE) is a volatile halogenated short-chain hydrocarbon used in dry cleaning, metal cleaning, the synthesis of other chemicals, and household products such as water repellants, silicone lubricants, and spot removers (ATSDR 2014). Both EPA and the Department of Health and Human Services (DHHS) consider tetrachloroethylene to be a likely human carcinogen (ATSDR 2014).

Everyone is exposed to low levels of radium in the air, water, and food. Higher levels may be found in drinking water from groundwater wells (EPA 2019). Exposure to higher levels of radium over a long period of time may result in harmful effects including anemia, cataracts, fractured teeth, and bone, liver, and breast cancer and death (ATSDR 1999; EPA 2019).

Trichloroethylene (TCE) is a volatile halogenated hydrocarbon used primarily as an industrial degreaser and solvent, and in the synthesis of other chemicals (ATSDR 2016). Exposure to TCE in drinking water is associated with kidney cancer, non-Hodgkin lymphoma, and cardiac birth defects (ATSDR 2016). A recent EPA toxicological review characterized TCE as carcinogenic in humans by all routes of exposure (EPA 2011d).

Uranium is a naturally occurring radioactive element found in food, air, and water as part of the natural environment (ATSDR 2013). Variable concentrations of uranium occur naturally in drinking water sources. In some locations, the natural concentrations may have increased due to mining and milling of uranium. After long-term or repeated exposure, the kidneys, liver, and bones can accumulate uranium, with the largest amounts being stored in the bones (Li et al. 2005). Health outcomes that may occur with uranium overexposure, based on both observed human effects and animal studies, include non-malignant respiratory disease (fibrosis, emphysema) and impaired kidney function and nephrotoxicity (Kurttio et al. 2006; Arzuaga et al. 2010; EPA 2010).

This surveillance summary describes the current available datasets for community drinking water and characterizes those data to provide meaningful estimates of drinking water quality in 24 states.

## Methods

### Data collection

Health departments in states funded by the Tracking Program (recipients) extract sample level drinking water data from their state SDWIS (Safe Drinking Water Information System) and calculate an annual mean and maximum concentration for each of the 10 contaminants (arsenic, nitrates, disinfection byproducts (total trihalomethanes (TTHM) and haloacetic acids (HAA5)), atrazine, DEHP (di (2-ethylhexyl) phthalate), trichloroethylene (TCE), perchloroethylene (PCE), uranium, and radium) for each CWS sampled at least once during the year.

A CWS is not necessarily sampled every year for each contaminant, or it may be sampled more than once during a year depending on the regulatory requirements. For nitrate, arsenic, atrazine, radium, uranium, DEHP, TCE, and PCE, average concentration values are derived from first averaging by sampling station, then averaging by CWS. For disinfection-by-products (TTHM and HAA5), annual average concentration values are derived from first averaging by day, then by CWS. Maximums for all 10 analytes are derived by taking the annual maximum for each CWS. The concentration data are submitted along with a summary file that describes the CWS for twenty-four states: California (CA), Colorado (CO), Connecticut (CT), Florida (FL), Iowa (IA), Kansas (KS), Kentucky (KY), Louisiana (LA), Massachusetts (MA), Maryland (MD), Maine (ME), Minnesota (MN), Missouri (MO), New Hampshire (NH), New Jersey (NJ), New Mexico (NM), New York (NY), Oregon (OR), Pennsylvania (PA), South Carolina (SC), Utah (UT), Vermont (VT), Washington (WA), and Wisconsin (WI). Data are validated by Tracking Program staff to ensure quality and completeness.

The drinking water data are available for most recipients from 2000 to 2010. The data availability for each contaminant during 2000–2010 and number of CWS with the total population served by those CWS for each state are summarized in Appendix 1 (Tables 1 and 2).

**Table 1:** Median number and percent of Community Water Systems that have a maximum concentration of contaminant that exceeds the MCL, 2000–2010

State	Arsenic	%	HAA5	%	Nitrate	%	Radium	%	TCE	%	TTHM	%	Uranium	%	Number of CWS
CA	*87*	*2.9*	*		*66*	*2.2*	0	0.0	*12*	*0.4*	*		*34*	1.1	3016
CO	6	0.6	32	3.3	5	0.5	*22*	*2.3*	0	0.0	32	3.3	*12*	*1.3*	963
CT	6	1.1	12	2.2	0	0.0	4	0.7	2	0.4	19	3.4	8	*1.4*	558
FL	0	0.0	*83*	4.9	4	0.2	8	0.5	0	0.0	*123*	*7.2*	0	0.0	1702
IA	9	0.8	13	1.2	14	*1.3*	*18*	1.7	0	0.0	41	3.8	1	0.1	1077
KS	9	1.0	43	*5.0*	*21*	*2.4*	5	0.6	0	0.0	48	5.6	7	0.8	863
KY	0	0.0	*86*	*18.5*	0	0.0	0	0.0	0	0.0	*82*	*17.7*	0	0.0	464
LA	5	0.5	33	3.2	*		*		0	0.0	65	6.4	0	0.0	1023
MA	6	1.1	17	3.2	1	0.2	2	0.4	0	0.0	44	*8.2*	4	0.7	539
MD	12	*2.9*	7	1.7	2	0.5	4	1.0	0	0.0	12	3.0	1	0.3	408
ME	15	*4.0*	16	4.2	0	0.0	0	0.0	0	0.0	19	5.0	*		379
MN	15	1.6	3	0.3	1	0.1	*19*	*2.0*	0	0.0	8	0.8	0	0.0	962
MO	5	0.4	*64*	*5.1*	0	0.0	*27*	*2.2*	1	0.1	80	*6.4*	0	0.0	1244
NH	14	2.0	6	0.9	0	0.0	3	0.4	0	0.0	15	2.1	*15*	*2.1*	710
NJ	2	0.3	14	2.3	2	0.3	18	*3.0*	0	0.0	31	5.1	4	0.7	608
NM	*24*	*4.1*	9	1.5	2	0.3	2	0.3	0	0.0	15	2.6	*14*	*2.4*	587
NY	3	0.1	*55*	2.1	8	0.3	14	0.6	*15*	*0.6*	*102*	4.0	1	0.0	2578
OR	16	1.9	9	1.1	2	0.2	0	0.0	0	0.0	10	1.2	1	0.1	855
PA	*20*	1.0	51	2.5	*18*	0.9	*		2	0.1	*110*	5.3	2	0.1	2064
SC	1	0.2	20	3.3	0	0.0	8	1.3	0	0.0	27	4.5	2	0.3	605
UT	0	0.0	0	0.0	0	0.0	0	0.0	0	0.0	0	0.0	0	0.0	456
VT	2	0.5	37	*8.5*	0	0.0	4	0.9	0	0.0	24	5.5	1	0.2	434
WA	*62*	*2.7*	10	0.4	*26*	*1.1*	0	0.0	0	0.0	14	0.6	2	0.1	2340
WI	7	0.7	2	0.2	5	0.5	3	0.3	2	0.2	4	0.4	0	0.0	1068
Totals	326		622		177		161		34		925		109		25,503

*not collected

**Table 2:** Median population served by Community Water Systems that have a maximum concentration of contaminant that exceeds the MCL, 2000–2010

State	Arsenic	%	HAA5	%	Nitrate	%	Radium	%	TCE	%	TTHM	%	Uranium	%	Population served by CWS
CA	*931,189*	*2.3*	*		*1,732,973*	*4.2*	0	0.0	*624,664*	*1.5*	*		*387,100*	*0.9*	41,032,941
CO	13,792	0.2	252,553	4.2	2,422	0.0	7524	0.1	0	0.0	338,464	5.6	15,830	0.3	6,009,037
CT	39,410	1.5	576,824	21.7	0	0.0	936	0.0	20,080	0.8	678,105	25.5	3,038	0.1	2,664,364
FL	0	0.0	*1,059,501*	5.5	2310	0.0	9999	0.1	0	0.0	*2,931,097*	15.2	0	0.0	19,227,648
IA	10,466	0.4	40,367	1.5	1405	0.1	50,288	1.9	0	0.0	302,899	11.2	294	0.0	2,698,490
KS	8985	0.3	309,198	11.6	29,045	1.1	4,971	0.2	0	0.0	325,559	12.3	7759	0.3	2,658,459
KY	0	0.0	*1,214,537*	*26.8*	0	0.0	0	0.0	0	0.0	1,293,420	*28.5*	0	0.0	4,532,689
LA	5914	0.1	275,296	5.6	*		*		0	0.0	470,680	9.6	0	0.0	4,903,486
MA	8,423	0.1	798,137	9.4	0	0.0	235	0.0	0	0.0	*2,950,610*	*34.7*	2990	0.0	8,510,788
MD	45,360	0.9	462,444	9.0	300	0.0	*250,187*	*4.9*	0	0.0	*2,230,344*	*43.6*	5100	0.1	5,119,581
ME	8442	1.3	114,986	17.3	0	0.0	0	0.0	0	0.0	78,447	11.8	*		665,484
MN	45,236	1.1	4666	0.1	40	0.0	*174,506*	*4.1*	0	0.0	87,594	2.1	0	0.0	4,235,789
MO	15,393	0.3	358,715	7.7	0	0.0	56,963	1.2	3,718	0.1	440,849	9.4	0	0.0	4,670,953
NH	11,603	1.4	70,353	8.2	0	0.0	473	0.1	0	0.0	117,625	13.8	2,795	0.3	855,359
NJ	17,208	0.2	*1,099,444*	12.2	880	0.0	*341,314*	*3.8*	0	0.0	1,955,510	21.7	7,509	0.1	9,016,086
NM	*186,449*	*10.2*	46,724	2.6	3,856	0.2	433	0.0	0	0.0	74,627	4.1	18,345	1.0	1,830,248
NY	2,155	0.0	*7,041,428*	*42.8*	*126,561*	0.8	3,507	0.0	*800,885*	*4.9*	1,233,406	7.5	313	0.0	16,462,686
OR	5,547	0.2	94,903	2.5	1740	0.1	0	0.0	0	0.0	55,934	1.5	28	0.0	3,791,401
PA	42,265	0.4	*3,177,088*	*29.5*	18,808	0.2	*		2,500	0.0	*5,028,818*	*46.6*	3365	0.0	10,781,463
SC	49	0.0	498,444	12.9	0	0.0	4,113	0.1	0	0.0	382,209	9.9	1250	0.0	3,860,152
UT	0	0.0	0	0.0	0	0.0	0	0.0	0	0.0	0	0.0	0	0.0	2,691,364
VT	4766	1.1	115,855	*26.1*	0	0.0	509	0.1	0	0.0	95,201	21.5	139	0.0	443,471
WA	68,785	1.2	79,669	1.4	5686	0.1	0	0.0	0	0.0	69,593	1.3	199	0.0	5,576,823
WI	5270	0.1	23,501	0.6	4877	0.1	5,629	0.1	35,908	0.9	4749	0.1	0	0.0	3,992,015
Totals	1,476,707		17,714,633		1,930,903		911,587		1,487,755		21,145,740		456,054		166,230,777

*not collected

### Data analysis

Using the data provided by recipients, we identified annual mean and annual maximum concentrations greater than the MCL for each contaminant. We calculated the number and percent of CWS with an annual mean or an annual maximum concentration greater than the MCL for each contaminant for each state and for each year. We also calculated the number and percent of population served by CWS with annual mean concentration or annual maximum concentration greater than the MCL for each contaminant for each state and for each year. We then identified the median number and percent of those measures to represent the 11 year time period to produce an annual median number and percentage of CWS and population served by CWS that had a mean or a maximum concentration of contaminant that exceeded the MCL. We also calculated the number and percent of CWS with exceedances of the MCL by size to determine if a greater proportion of exceedances were seen at smaller systems.

SAS 9.3 (SAS Institute, Cary, North Carolina) was used to access and analyze the community drinking water data on the CDC tracking server.

## Results

Contaminants that occur more frequently in CWS with maximum concentrations greater than the MCL include the disinfection byproducts TTHM and HAA5, arsenic, nitrate, radium, and uranium (Table 1). The median annual number of CWS with annual maximum concentrations above the MCL for TTHM were 925 (4.1%) in any given year from 2000 to 2010; for HAA5, 622 (2.8%); arsenic, 326 (1.3%); nitrate, 177 (0.7%); radium, 161 (0.7%); and uranium, 109 (0.4%). The median number of CWS that have an annual maximum concentration that exceeds the MCL for atrazine, DEHP, PCE, and TCE is very low for most states. There was a median annual number of 8 CWS (0.03%) that exceeded for atrazine, 19 (0.08%) for DEHP, 38 (0.15%) for PCE, and 34 for TCE (0.13%).

The median number and percent of CWS that have a maximum concentration of contaminant that exceeds the MCL for years 2000–2010 are presented in Table 1. The greatest number and percent of CWS with median annual maximum concentrations exceeding the MCL for each contaminant for each state are shown in italics.

The median population and percent of population served by CWS with a maximum concentration of contaminant exceeding the MCL for years 2000–2010 for each grantee state is presented in Table 2. The highest numbers and percentages of populations with contaminants exceeding the MCL are shown in italics.

Tables 3 and 4 provide the 11-year median number and percentages of CWS and population served by CWS with mean concentrations of contaminant that exceeds the MCL for that contaminant. When considering the mean rather than the maximum, as anticipated, the numbers and percentages of exceedances greater than the MCL are fewer. For annual mean concentrations, the analytes that exceed the MCL most often are arsenic, the disinfection byproducts TTHM and HAA5, uranium, radium, and nitrate. The highest numbers and percentages of CWS and populations and percentages of populations served by CWS are highlighted in italics in both Tables 3 and 4.

**Table 3:** Median number and percent of Community Water Systems that have a mean concentration of contaminant that exceeds the MCL, 2000–2010

State	Arsenic	%	HAA5	%	Nitrate	%	Radium	%	TCE	%	TTHM	%	Uranium	%	Number of CWS
CA	*66*	*2.2*	*		*24*	*0.8*	0	0.0	1	0.0	*		*23*	*0.8*	3016
CO	5	0.5	4	0.4	2	0.2	*18*	*1.9*	0	0.0	4	0.4	*9*	*0.9*	963
CT	6	1.1	2	0.4	0	0.0	2	0.4	0	0.0	1	0.2	3	0.5	558
FL	0	0.0	*28*	1.7	1	0.1	0	0.0	0	0.0	*38*	*2.2*	0	0.0	1702
IA	6	0.6	2	0.2	2	0.2	*12*	*1.1*	0	0.0	5	0.5	1	0.1	1077
KS	6	0.7	*29*	*3.4*	*10*	*1.2*	3	0.4	0	0.0	*25*	*3.0*	5	0.6	863
KY	0	0.0	12	*2.6*	0	0.0	0	0.0	0	0.0	5	1.1	1	0.2	464
LA	5	0.5	14	1.4	*		*		0	0.0	*36*	*3.5*	0	0.0	1023
MA	3	0.6	1	0.2	0	0.0	0	0.0	0	0.0	5	1.0	3	0.6	539
MD	7	1.7	1	0.3	0	0.0	1	0.3	0	0.0	2	0.5	0	0.0	408
ME	9	*2.4*	4	1.1	0	0.0	0	0.0	0	0.0	9	2.4	*		379
MN	12	1.3	1	0.1	0	0.0	*14*	*1.5*	0	0.0	2	0.2	0	0.0	962
MO	2	0.2	*23*	1.9	0	0.0	*22*	*1.8*	1	0.1	*33*	*2.7*	0	0.0	1244
NH	5	0.7	2	0.3	0	0.0	3	0.4	0	0.0	3	0.4	*12*	*1.7*	710
NJ	0	0.0	0	0.0	1	0.2	6	1.0	0	0.0	1	0.2	2	0.3	608
NM	*21*	*3.6*	3	0.5	1	0.2	2	0.3	0	0.0	4	0.7	7	1.2	587
NY	2	0.1	13	0.5	1	0.0	9	0.4	*8*	*0.3*	22	0.9	0	0.0	2578
OR	14	1.6	2	0.2	0	0.0	0	0.0	0	0.0	2	0.2	0	0.0	855
PA	11	0.5	7	0.3	4	0.2	*		0	0.0	14	0.7	1	0.1	2064
SC	0	0.0	0	0.0	0	0.0	4	0.7	0	0.0	2	0.3	2	0.3	605
UT	0	0.0	0	0.0	0	0.0	0	0.0	0	0.0	0	0.0	0	0.0	456
VT	2	0.5	10	*2.3*	0	0.0	3	0.7	0	0.0	2	0.5	0	0.0	434
WA	*45*	*1.9*	2	0.1	*14*	*0.6*	0	0.0	0	0.0	8	0.3	2	0.1	2340
WI	3	0.3	1	0.1	1	0.1	3	0.3	0	0.0	2	0.2	0	0.0	1068
Totals	230		161		61		102		10		225		71		25,503

*not collected

**Table 4:** Median population served by Community Water Systems that have a mean concentration of contaminant that exceeds the MCL, 2000–2010

State	Arsenic	%	HAA5	%	Nitrate	%	Radium	%	TCE	%	TTHM	%	Uranium	%	Population served by CWS
CA	*173,439*	0.4	*		*14,232*	0.0	0	0.0	25	0.0	*		*8517*	0.0	41,032,941
CO	11,829	0.2	2121	0.0	197	0.0	4193	0.1	0	0.0	2115	0.0	*15,750*	*0.3*	6,009,037
CT	36,656	*1.4*	28,452	1.1	0	0.0	167	0.0	0	0.0	680	0.0	1121	0.0	2,664,364
FL	0	0.0	*100,769*	0.5	164	0.0	0	0.0	0	0.0	*71,781*	0.4	0	0.0	19,227,648
IA	3984	0.2	4321	0.2	932	0.0	17,628	0.7	0	0.0	13,691	0.5	294	0.0	2,698,490
KS	4304	0.2	*39,979*	*1.5*	*6595*	*0.3*	2373	0.1	0	0.0	39,868	*1.5*	4582	0.2	2,658,459
KY	0	0.0	*53,873*	1.3	0	0.0	0	0.0	0	0.0	17,376	0.4	0	0.0	4,532,689
LA	2185	0.0	25,972	0.5	*		*		0	0.0	*181,350*	*3.7*	0	0.0	4,903,486
MA	1440	0.0	13,000	0.2	0	0.0	0	0.0	0	0.0	32,320	0.4	1325	0.0	8,510,788
MD	6310	0.1	3672	0.1	0	0.0	150	0.0	0	0.0	7040	0.1	0	0.0	5,119,581
ME	1568	0.2	21,513	*3.2*	0	0.0	0	0.0	0	0.0	12,971	*2.0*	*		665,484
MN	4264	0.1	364	0.0	0	0.0	*73,078*	*1.7*	0	0.0	2027	0.1	0	0.0	4,235,789
MO	7700	0.2	37,136	0.8	0	0.0	*44,111*	*0.9*	350	0.0	*66,721*	*1.4*	0	0.0	4,670,953
NH	589	0.1	2600	0.3	0	0.0	236	0.0	0	0.0	4924	0.6	1365	0.2	855,359
NJ	0	0.0	0	0.0	81	0.0	*66,698*	*0.7*	00	0.0	12,575	0.1	89	0.0	9,016,086
NM	*109,336*	*6.0*	3,762	0.2	120	0.0	315	0.0	0	0.0	3122	0.2	*5725*	*0.3*	*1,830,248*
NY	193	0.0	54,513	0.3	150	0.0	1448	0.0	*304,562*	*1.9*	*58,501*	0.4	0	0.0	16,462,686
OR	4446	0.1	2501	0.1	0	0.0	0	0.0	00	0.0	3945	0.1	0	0.0	3,791,401
PA	14,647	0.1	14,722	0.1	344	0.0	*		0	0.0	37,516	0.4	1731	0.0	10,781,463
SC	0	0.0	0	0.0	0	0.0	3648	0.1	0	0.0	1914	0.1	1200	0.0	3,860,152
UT	0	0.0	0	0.0	0	0.0	0	0.0	0	0.0	0	0.0	0	0.0	2,691,364
VT	457	0.1	25,903	*5.8*	0	0.0	437	0.0	0	0.0	2162	0.5	0	0.0	443,471
WA	19,084	0.3	5266	0.1	1853	0.0	0	0.0	0	0.0	5598	0.1	124	0.0	5,576,823
WI	311	0.0	95	0.0	40	0.0	5629	0.1	0	0.0	2639	0.1	0	0.0	3,992,015
Totals	402,742		440,534		24,708		220,111		304,937		580,836		41,823		166,230,777

*not collected

Table 5 provides a breakdown of the number of annual means and annual maximums that exceeded the MCL by size of CWS for the 11-year time period. Eighty-three percent of the annual means and 85% of the annual maximums that exceeded the MCL occurred at very small and small CWS, which serve 25–500 and 501 to 3,300 people year-round. The greatest percentage of exceedances for both mean (46%) and maximum (51%) concentrations above the MCL occurred at small CWS.

**Table 5:** Size of Community Water System and annual mean and annual maximum concentrations of contaminants greater than the MCL (2000–2010)

Size of CWS (based on population served)	Median # of CWS w/annual means > MCL (% of systems at that size category)	Median # of CWS w/annual maximums > MCL (% of systems at that size category)
Very small (< 500)	4191 (28%)	5418 (37%)
Small (501–3300)	2541 (46%)	2809 (51%)
Medium (3301–10,000)	807 (39%)	861 (41%)
Large (10,001–100,000)	534 (27%)	601 (31%)
Very large (100,001+)	41 (16%)	44 (17%)
Total	8114 (33%)	9733 (40%)

Table 6 shows the source of water by size of CWS. Very small and small CWS serving between 25 and 3,300 people use groundwater (GWU and GW) as a primary source of drinking water. Ninety-one percent of very small CWS and 76% of small CWS use groundwater as a drinking water source; combined groundwater is used as a drinking water source by 87% of the very small and small CWS (Table 6).

**Table 6:** Primary water source for Community Water Systems by size (2010)

Size of CWS (based on population served)	SW	GW	GWU	Unknown	Total
Very small (< 500)	1262	13,595	178	212	15,247
Small (501 to 3300)	1329	4,322	101	120	5872
Medium (3301 to 10,000)	803	1219	27	99	2148
Large (10,001 to 100,000)	922	833	21	202	1978
Very large (> 100,000)	126	54	3	75	258
Total	4,442	20,023	330	710	25,503

*SW* surface water; *GW* groundwater; *GWU* groundwater under the influence of surface water

## Discussion

Annual maximum concentrations of the drinking water contaminants represent a worst-case exposure to those contaminants. Exposure to those maximum concentrations is not continuous, but it provides information on how high the occasional spike of contaminant is for CWS and populations receiving drinking water from those CWS. Annual mean concentration data provide insight into the central tendency of the data to exceed the MCL and represent the chronic exposure to contaminants. The total number of CWS that have an annual maximum concentration of contaminant that exceeds the MCL is far greater for TTHM (925) and HAA5 (622) than for arsenic (326) (Table 1). In contrast, the total number of CWS that have an annual mean concentration of contaminant that exceeds the MCL is highest for arsenic (230), followed closely by TTHM (225) and then HAA5 (161). Similarly, the total number of CWS that have an annual maximum concentration of contaminant that exceeds the MCL is greater for nitrate (177) than for radium (161) and uranium (109). The annual mean concentration of contaminant that exceeds the MCL is higher for radium (102) and uranium (71) than for nitrate (61) (Table 1). A possible and plausible reason for these differences may be due to the nature of the contaminants. Arsenic, radium, and uranium are naturally occurring elements found in bedrock. They are conservative in nature, and their concentrations do not change very much between sampling events. Although arsenic has been used in industrial and agricultural applications, the concentrations found in drinking water can usually be attributed to natural sources. TTHM and HAA5, the DBPs, are created from the interaction between disinfectants and organic matter in the water during drinking water disinfection. Since the need for disinfection varies over time and the amount of organic material in the water varies, DBPs in drinking water are also seen to vary greatly—seasonally (Singer et al. 1981; Whitaker et al. 2003; Rodriguez et al. 2004), higher during the month of warmest weather; spatially (Rodriguez et al. 2004) and temporally (Chen and Weisel 1998; Rodriguez and Serodes 2001). This variation is also why DBPs are sampled more frequently than arsenic, radium, or uranium, but quarterly or triennial sampling may still not provide a good estimate of an annual average exposure (Singer et al. 1981; Whitaker et al. 2003, Rodriguez et al. 2004). Nitrate in drinking water varies seasonally, as the variation in runoff from fertilizer use, or the land application of animal wastes changes from one season to the next. Nitrate is also sampled quarterly, but similar to DBPs, the variation is not likely to be captured by this frequency of sampling. Since DBPs and nitrate can spike during different times of the year, their maximum concentrations could be expected to be higher than the naturally occurring elements, arsenic, radium, and uranium.

CA has the greatest number of people served by CWS and the greatest number of CWS, and so it had higher numbers of CWS with maximum and mean concentrations of arsenic, nitrate, and uranium that exceeded the MCL (CA does not collect or submit data for DBPs). On a percentage basis, other states had higher percentages of CWS exceed the MCL for maximum and mean concentrations of contaminants, e.g., NM had the highest percentage of CWS with a maximum and a mean concentration of arsenic exceeding the MCL, and a maximum concentration of uranium that exceeded the MCL. KY had the greatest percentage of CWS with maximum concentrations of HAA5 and TTHM exceeding the MCL; KS had a higher percentage of CWS with a maximum and mean concentration of nitrate that exceeded the MCL. For radium, NJ had the highest percentages of CWS exceed the annual maximum concentrations, while CO had the highest percentage of CWS with mean concentrations that exceeded the MCL.

Those states consistently in the high 4 to 5 states with the greatest number of exceedances of the MCL for TTHM, HAA5, arsenic, nitrate, radium, and uranium for all years are CA and PA for the annual maximum and CA, MO, and FL for the annual mean. Those states consistently in the high 4 to 5 states with greatest percentage of exceedances were MO for the maximum and KS and MO for the mean. We looked to see if those states had a greater number and percentage of very small and small CWS, where problems with non-compliance are more common, but that was not the case.

Less than 5% of the CWS in 24 states have annual maximum concentrations of contaminants that exceed the MCL for 8 out of 10 of the contaminants examined. Exceptions to this were TTHM and HAA5, which occurred in excess of the MCL more frequently, e.g., in KY, 18% of CWS exceeded the MCL for both TTHM and HAA5; six other states (FL, KS, LA, MA, MO, and VT) had greater than 5% of CWS exceed the MCL for TTHM; two states in addition to KY (MO and VT) had greater than 5% of CWS exceed the MCL for HAA5. In terms of population served, NM had 10% of their population served receive drinking water with arsenic above the MCL. Fifteen of 24 states had more than 5% of their population served receive drinking water with exceedances of the MCL for HAA5. There were 17 of 24 states that had higher than 5% of their population served receive drinking water with exceedances of the MCL for TTHM.

We did anticipate seeing the greatest number of non-compliant records at smaller CWS. EPA cites 8 challenges that small systems face in achieving compliance, and three of them are financial (EPA 2016). Lack of financial resources, aging infrastructure, difficulties obtaining financial assistance, cost of scale, management limitations, lack of long term planning, system operator issues, and challenges with understanding and/or complying with regulations are the reasons EPA cites as issues in achieving compliance (EPA 2016).

The results provided in this surveillance report are subject to limitations. The concentrations reported represent population-level exposures, and individual exposures may be different, e.g., contaminant concentrations in distribution lines will differ and filtration at the tap will remove or reduce concentrations of some contaminants. Not all states collect all ten of the drinking water contaminants, e.g., CA does not sample for DBPs, PA does not sample for radium, and LA does not sample for nitrate. Monitoring frequency is less for states that have shown lower or non-detectable concentrations of some contaminants (i.e., arsenic, radium) therefore, sometimes concentration data from states is sparse, with few samples that span years of non-testing.

Strengths of the data include the high number of records, from 24 states, 11 years of data, with a high degree of quality assurance and quality control.

## Conclusion

Exposure to arsenic and disinfection byproducts in community drinking water constitutes the greatest potential non-compliant contaminant exposure to the populations in the 24 states examined, during the time period 2000–2010. Nitrate, radium, and uranium with annual maximum concentrations greater than the MCL potentially exposed less than 5% of the population served in the states with the greatest concentrations over the 11-year period. Very small and small CWS are the systems’ greatest difficulty in achieving compliance. Concentrating on public health interventions at these smaller systems for those specific drinking water contaminants may achieve the greatest public health improvement.

Federal, state, and local public health agencies can use tracking data on community drinking water for surveillance purposes to estimate trends over time and to design public health actions to targeted areas to reduce contaminants among at-risk populations.

## Appendix 1

**Table 7:** Years of available data for each contributing state, 2010–2010

State	Arsenic	Atrazine	DEHP	HAA5	Nitrate	PCE	Radium	TCE	TTHM	Uranium
CA	2000–2010	2000–2010	2000–2010	No data	2000–2010	2000–2010	2000–2010	2000–2010	No data	2000–2010
CO	2000–2010	2000–2010	2000–2010	2002–2010	2000–2010	2000–2010	2000–2010	2000–2010	2000–2010	2000–2010
CT	2001–2010	2002–2010	2002–2010	2000, 2002–10	2000–2010	2000–2010	2001–2010	2000–2010	2000–2010	2001–2010
FL	2000–2010	2000–2010	2000–2010	2000–2010	2000–2010	2000–2010	2000–2010	2000–2010	2000–2010	2000–2010
IA	2000–2010	2000–2010	2000–2010	2000–2010	2000–2010	2000–2010	2000–2010	2000–2010	2000–2010	2000–2010
KS	2000–2010	2000–2010	2000–2010	2000–2010	2000–2010	2000–2010	2002–2010	2000–2010	2000–2010	2000–2010
KY	2000–2010	2000–2010	2000–2010	2002, 2004–2010	2000–2010	2000–2010	2002–2010	2000–2010	2000–2010	2006–2010
LA	2000–2010	2000–2010	2000–2010	2003–2010	No data	2000–2010	No data	2000–2010	2002–2010	2002, 2008–2010
MA	2000–2010	2000–2010	2000–2010	2000–2010	2000–2010	2000–2010	2000–2010	2000–2010	2000–2010	2000–2010
MD	2000–2010	2000–2010	No data	2002–2010	2000–2010	2000–2010	2000–2010	2000–2010	2000–2010	2000–2010
ME	2000–2010	2000–2010	2000–2010	2000–2010	2000–2010	2000–2010	2000–2010	2000–2010	2000–2010	No data
MN	2000–2010	2000–2010	2000–2010	2003–2010	2000–2010	2000–2010	2000–2010	2000–2010	2003–2010	2000–2010
MO	2000–2010	2000–2010	2003, 2005, 2009–2010	2000–2010	2000–2010	2000–2010	2002–2010	2000–2010	2000–2010	2000–2010
NH	2000–2010	2000–2010	2000–2010	2002–2009	2000–2010	2000–2010	2000, 2002–2010	2000–2010	2002–2009	2000–2010
NJ	2000–2010	2000–2010	2000–2001, 2004, 2006–10	2001–2010	2000–2010	2000–2010	2002–2010	2000–2010	2000–2010	2001–2010
NM	2000–2010	2000–2010	2000–2010	2002–2010	2000–2010	2000–2010	2002–2010	2000–2010	2000–2010	2000–2001, 2003–2010
NY	2000–2010	2000–2010	2000–2010	2000–2010	2000–2010	2000–2010	2000–2010	2000–2010	2000–2010	2001–2010
OR	2002–2010	2002–2010	2002–2010	2002–2010	2002–2010	2002–2010	2002–2010	2002–2010	2002–2010	2002–2010
PA	2000–2010	2000–2010	2000–2010	2000–2010	2000–2010	2000–2010	No data	2000–2010	2000–2010	2001–2010
SC	2003, 2005–10	2002–2010	No data	2004–2010	2002–2010	2005–2010	2004–2010	2005–2010	2004–2010	2007–2010
UT	2000–2010	2000–2010	2000–2010	2004–2010	2004–2010	2000–2010	2005, 2008–2010	2000–2010	2004–2010	2000–2010
VT	2000–2010	2000–2010	2000–2010	2000–2010	2000–2010	2000–2010	2002–2010	2000–2010	2000–2010	2000–2010
WA	2000–2010	2000–2010	2000–2010	2000–2010	2000–2010	2000–2010	2001, 2003–2010	2000–2010	2000–2010	2000–2010
WI	2000–2010	2000–2010	2000–2010	2002–2010	2000–2010	2000–2010	2000–2010	2000–2010	2000–2010	2000–2002, 2005–2010

**Table 8.** Number of Community Water Systems (CWS) per State and the Population served by those CWS (2010)

State	Number of CWS	Population served by CWS
CA	3016	41,032,941
CO	963	6,009,037
CT	558	2,664,364
FL	1702	19,227,648
IA	1077	2,698,490
KS	863	2,658,459
KY	464	4,532,689
LA	1023	4,903,486
MA	539	8,510,788
MD	408	5,119,581
ME	379	665,484
MN	962	4,235,789
MO	1244	4,670,953
NH	710	855,359
NJ	608	9,016,086
NM	587	1,830,248
NY	2578	16,462,686
OR	855	3,791,401
PA	2064	10,781,463
SC	605	3,860,152
UT	456	2,691,364
VT	434	443,471
WA	2340	5,576,823
WI	1068	3,992,015
Total	25,503	166,230,777

Table 8 lists the number of community water systems in each tracking state with the total population served by those community water systems. CA has the greatest number of CWS, 3016 and serves the greatest number of people: 41,032,941. ME has the lowest number of CWS with 379, which serve a population of 665,484 people. VT has a greater number of CWS than ME (434 v. 379) but they serve fewer people (443,471 v. 665,484). In a similar manner, NY has the second highest number of CWS with 2578, but they serve a smaller population (16,462,686) than FL with 1702 CWS serving 19,227,648 people.
